# A Canonical Circuit for Generating Phase-Amplitude Coupling

**DOI:** 10.1371/journal.pone.0102591

**Published:** 2014-08-19

**Authors:** Angela C. E. Onslow, Matthew W. Jones, Rafal Bogacz

**Affiliations:** 1 Bristol Centre for Complexity Sciences (B.C.C.S.), University of Bristol, Queen's Building, Bristol, United Kingdom; 2 Department of Psychology, Center for Memory and Brain, Boston University, Boston, MA, United States of America; 3 School of Physiology and Pharmacology, University of Bristol, Medical Sciences Building, Bristol, United Kingdom; 4 Department of Computer Science, University of Bristol, Merchant Venturers Building, Bristol, United Kingdom; 5 Nuffield Department of Clinical Neurosciences, University of Oxford, John Radcliffe Hospital, Oxford, United Kingdom; 6 Medical Research Council Anatomical Neuropharmacology Unit, Department of Pharmacology, University of Oxford, Oxford, United Kingdom; Federal University of Rio Grande do Norte, Brazil

## Abstract

‘Phase amplitude coupling’ (PAC) in oscillatory neural activity describes a phenomenon whereby the amplitude of higher frequency activity is modulated by the phase of lower frequency activity. Such coupled oscillatory activity – also referred to as ‘cross-frequency coupling’ or ‘nested rhythms’ – has been shown to occur in a number of brain regions and at behaviorally relevant time points during cognitive tasks; this suggests functional relevance, but the circuit mechanisms of PAC generation remain unclear. In this paper we present a model of a canonical circuit for generating PAC activity, showing how interconnected excitatory and inhibitory neural populations can be periodically shifted in to and out of oscillatory firing patterns by afferent drive, hence generating higher frequency oscillations phase-locked to a lower frequency, oscillating input signal. Since many brain regions contain mutually connected excitatory-inhibitory populations receiving oscillatory input, the simplicity of the mechanism generating PAC in such networks may explain the ubiquity of PAC across diverse neural systems and behaviors. Analytic treatment of this circuit as a nonlinear dynamical system demonstrates how connection strengths and inputs to the populations can be varied in order to change the extent and nature of PAC activity, importantly which phase of the lower frequency rhythm the higher frequency activity is locked to. Consequently, this model can inform attempts to associate distinct types of PAC with different network topologies and physiologies in real data.

## Introduction

There is a growing body of evidence demonstrating that oscillatory activity at various scales within the brain is correlated with behavior in a task-dependent manner [1]–[7]. This has prompted the hypothesis that oscillatory activity may be produced and dynamically modulated by the nervous system in order to effectuate various executive functions [8]–[14]. Oscillatory neural activity is traditionally binned into several commonly occurring frequency bands that appear to predominate during particular behaviors [1]. These different frequencies can co-occur and there is increasing interest in how activities occurring at two different frequencies dynamically modulate one another [15], producing a form of coupling between oscillations of different frequencies that could allow for the integration of information across multiple spatial and temporal scales [16], [17]. It has been suggested that a hierarchy of interacting oscillations could segment the conscious experience into discrete, serial processing windows [18]–[21]; for example, consecutively visited places in an environment might be encoded as the ordered firing of place cells relative to a theta cycle oscillation in the hippocampus, in order to maintain conscious awareness of current position [22]. The sequential order of memorized items in a list might be encoded in a similar way [23], [24].

Coupling between different frequencies of neural activity can take three forms: phase-phase coupling, amplitude-amplitude coupling and phase-amplitude coupling [9], [15]. The latter is the focus of the modeling work presented here; we will demonstrate, using a canonical circuit consisting of excitatory and inhibitory neural populations, how phase-amplitude coupling (referred to henceforth as PAC) can occur and how the particular type of PAC can be manipulated by varying the model parameters.

PAC is said to occur when the amplitude envelope of a high-frequency oscillation varies with the phase of a slower oscillation. The first recorded example of this type of coupling was found to occur between theta (4–12 Hz) and gamma (40–100 Hz) band activity in the hippocampus [25]–[28]. Theta-gamma PAC activity has also been recorded in human neocortex [29], occipital and frontal regions [30], [31] and medial temporal lobe [32]; it has also been found to occur between various regions involved in auditory attentional control in humans [33]. These studies have shown behavior-related changes in PAC during short-term memory, working memory and word recognition tasks. There are also reports of PAC occurring between various other frequency band combinations [16], [17], [30], [34], [35]; whatever the constituent frequency combinations, PAC tends to occur most strongly during cognitively demanding epochs of tasks.

Studies of theta-gamma PAC in rodents have been particularly illuminating regarding dynamic changes in PAC as well as its functional correlates. It has been shown that theta-gamma PAC can occur both within and between brain structures, for example within and between the hippocampus and the striatum; [36]. In this study PAC activity varied in strength from no coupling to strong coupling and back to no coupling over a period of a few seconds, with the strongest coupling occurring whilst the animal listened to a tone indicative of which turn to make in a maze-based task. Theta-gamma PAC in CA3 of the rodent hippocampus has been found to increase in strength during learning of an item-context association task [37] (a similar result has been demonstrated in inferotemporal cortex of sheep following learning [38]). Rodent studies have also revealed theta-gamma coupling occurring between hippocampal regions and entorhinal cortex; three different frequency bands within the gamma range have been found to couple to different phases of the hippocampal theta rhythm and the studies' authors suggest that these bands could provide multiple separate channels of communication between the two structures [39], [40]. There is also evidence that theta-gamma PAC occurring between hippocampus, prefrontal cortex and entorhinal cortex increases with learning [41].

Various single and multi-compartmental models that are able to produce theta-gamma PAC activity are reviewed in [42]. These models focus on modeling oscillations in the hippocampus, specifically how purely inhibitory and excitatory-inhibitory networks of cells can generate network gamma oscillations, how single neuron models can produce resonant theta oscillations (the role of oriens-lacunosum moleculare cells and the I_h_ and A currents appear to be particularly important) and how theta-nested gamma activity can arise from a combination of the two. The model of White et al. [43] uses a network of purely inhibitory Hodgkin-Huxley neurons of two types, one with long inhibitory post-synaptic potentials (IPSPs) and one with short IPSPs, to produce theta-gamma PAC activity (however this behavior is found to be un-robust in the absence of periodic input). Networks of excitatory and inhibitory integrate-and-fire (IF) neurons have also been used to generate theta-gamma coupled activity [23], [44] for the purpose of exploring possible functional mechanisms and how the strength of coupling might change during learning. The model of Zhang et al. [44] is similar in circuit structure to the “E-I-O” model presented by Kopell et al. [42] (E – excitatory, I – inhibitory, O - oriens-lacunosum moleculare cells, which are inhibitory and theta resonant) but uses networks of IF neurons for each of the constituent populations: an excitatory population, an inhibitory population with fast GABAergic currents responsible for generating gamma oscillations and an inhibitory population with slow GABAergic currents responsible for generating theta oscillations. The model of Lisman and Idiart [23] uses an imposed theta oscillation (which produces both depolarizing and hyperpolarizing effects), combined with the effects of an afterdepolarization potential, to periodically bring excitatory IF neurons above firing threshold, whilst gamma frequency rhythmicity of their spikes is ensured by inhibitory feedback.

The defining feature of these previous PAC models is that gamma frequency oscillations arise due to the choice of time constants involved in interactions between E-I or I-I populations of cells (such oscillatory behavior is typical of pyramidal-interneuron gamma (PING) [45]–[49] or interneuronal gamma (ING) [48], [49] networks). This gamma activity is then periodically inhibited by a theta rhythm, which is imposed by either an external source [23] or theta resonant cells within the network [42]–[44]. The topology of the model we will present here echoes this, with a network gamma rhythm being generated by the interaction of an E and an I population and theta frequency external input being received by either population. However, our model serves to illustrate that gamma activity is not required to be the default mode of the system in the absence of theta frequency input but can instead require some level of external input to arise. The magnitude of theta frequency input can act as a bifurcation parameter that moves the system into a gamma-oscillation-producing regime. If the range of input values that produce gamma oscillations in the system is bounded, as in our model, then it is possible to use the input to control which phase of theta the gamma oscillations are locked to: the peak, the trough or the ascending and descending phases.

Previous PAC models demonstrated coupling to the peak or the trough of the low frequency rhythm but not to the ascending and descending phases. There is empirical evidence that high frequency activity may be coupled not just to the peak of the low frequency oscillation [50], [51] but also to the trough [29], [36], [52] and to the ascending [36] and descending phases separately [39], [50], [51]. It is postulated that coupling of gamma activity to different phases of theta oscillations within hippocampal CA1 could facilitate the interpretation of incoming information from two distinct channels (CA3 and entorhinal cortex) [39]. Our model can be used to produce coupling to the full variety of phases of the low frequency rhythm that have been observed empirically. We use a modified Wilson-Cowan firing rate model [53] to show how theta frequency input received by either the excitatory or the inhibitory population can move the system periodically in and out of the regime in which it produces intrinsic gamma frequency oscillatory activity. The result is a generally applicable model in which gamma band activity is produced at a particular phase of the theta frequency input.

## Methods

### Description of the model

**Figure 1:**
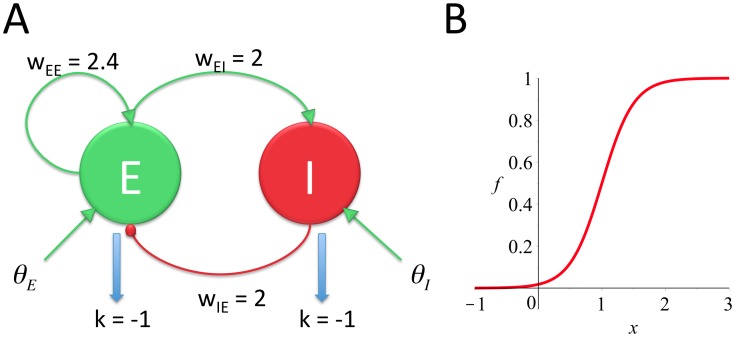
Diagrammatic representation of the PAC model (A) and the choice of sigmoidal response function (B). A: grey arrows represent excitatory connections (+), black circles represent inhibitory connections (−). All weights in the model are positive, excitatory or inhibitory connections appear as +/− signs in the model equations (Equation (1)). External input can be received by either the E or the I population. Mid-grey arrows represent the leak in activity levels as a result of passive membrane properties. B: the exact shape of the sigmoid chosen (Equation (2)). The mean threshold is at x = 1. β = 4.

In order to create a model to compare with empirical evidence of PAC occurring in local field potential recordings, we chose to model dynamics at the population level, in terms of the average firing rate of local populations of neurons. This approach follows the model introduced by Wilson & Cowan [53]. The model consists of a single excitatory and a single inhibitory neural population that are reciprocally connected (see Figure 1A). The excitatory population also sends a recurrent projection to itself. This recurrent connection is required for the model to be able to produce intrinsic oscillations (refer to [53]; we discuss the necessity of this assumption in the next section). Both populations experience some inherent leak in their activity levels as a result of the passive electrical properties of component neurons. Both populations also receive independent external inputs, assumed to be from other neural populations or brain regions. The activity of each population is modeled as a sigmoidal response function of the inputs to that population (Figure 1B and Equation (2)).

The model is described by the following continuous-time differential equations:
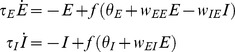
(1)Here *E* and *I* denote the average activity levels of excitatory and inhibitory populations. *θ_E_* and *θ_I_* are the external inputs to the two populations, E and I respectively. *w_EE_*, *w_EI_* and *w_IE_* are the weights on the various connections in the model and 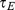 and 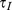 are characteristic time constants, which correspond physiologically to the membrane time constants of particular neural populations. In all the simulations presented in this paper these time constants were set such that what we will describe as the intrinsic frequency response of the system, when forced with external input *θ_E_* = 0.5, *θ_I_* = 0, is a 55 Hz (gamma) oscillation (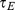 = 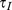 = 0.0032 s, equivalent to the natural frequency of the system (0.176 Hz) divided by the desired (gamma) frequency of 55 Hz). It should be kept in mind that the frequency of oscillations generated by the system is a function of all the model parameters, including the inputs *θ_E_* and *θ_I_* and will vary with these accordingly; however large variation in the frequency (>a few Hz) is only seen when parameter values are close to bifurcation points. The weight parameters also remain the same for all simulations that follow; *w_EE_* = 2.4, *w_EI_* = 2 and *w_IE_* = 2. These are sample parameter values for which the system can generate oscillations and the range of values of *θ_E_* and *θ_I_* that correspond to observing oscillations in the system is bounded. Extensive analysis of the Wilson-Cowan model for particular values of the weight parameters are given in [54], [55] and highlight the wide range of bifurcations and behaviour that are possible in the model.

The sigmoid response function *f*(*x*) was chosen as:
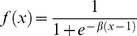
(2)The *β* parameter, which determines the steepness of the sigmoid function, was set equal to 4 in all the results that will be presented (this value results in the derivative of *f*(*x*) being equal to 1 at its steepest point). This choice of sigmoidal response function is slightly altered from that used in the original Wilson & Cowan model. Note that *f* (0)>0 (see Figure 1B), as opposed to *f* (0) = 0, which was employed by Wilson & Cowan to make the state corresponding to zero firing rate activity stable. Hence, even when a population receives zero input in our model it can still produce firing rate activity. We consider this modification biologically plausible since neurons within a population may produce spontaneous spiking.

### Conditions for generation of intrinsic oscillations

The topology shown in Figure 1A is capable of generating oscillations since the reciprocal connections between the E and the I populations form a canonical negative feedback circuit. If the activity of E increases so too does the activity of I, which then inhibits E and lowers its activity level. This in turn lowers I's activity level and if the system is correctly parameterized inhibition will be lowered sufficiently for E to increase its activity level again. This process repeats cyclically, producing oscillations in both E and I's activity levels. The addition of positive feedback from E to itself is intended to amplify E, allowing E to increase its activity level more rapidly than the activity of the I population can quench E and damp oscillations. The mathematical analysis that follows demonstrates the importance of this positive feedback connection (*w_EE_*) for the generation of oscillations in our model. We also use mathematical analysis to demonstrate how the external inputs to the model, *θ_E_* & *θ_I_*, affect the dynamical behavior and the generation of intrinsic oscillations.

Understanding when and how this system (Equation (1)) behaves as an oscillator is possible via analysis of the system's nullclines and their arrangement in the E, I phase plane. Oscillatory solutions of a system occur when there is a limit cycle present in the system's phase plane. The Poincaré-Bendixson theorem [56] says that a limit cycle must exist inside a trapping region (a region of the phase plane that all trajectories are attracted towards and cannot escape) if all the equilibria within that region are unstable. Trajectories that our system can take are subject to an inherent trapping region in the phase plane (E = [0, 1], I = [0, 1]), due to the limits of the populations' sigmoidal response functions. We will now use analysis of the system's nullclines to consider when a single unstable fixed point exists in the system's phase-plane and hence a limit cycle exists and the system behaves as an oscillator.

The nullclines of the system can be found by setting the derivatives in (1) to 0:
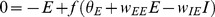
(3)

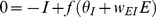
(4)We solve equations (3) and (4) for I to produce equations (5) and (6).
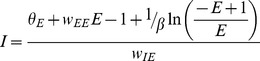
(5)

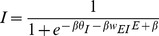
(6)


**Figure 2:**
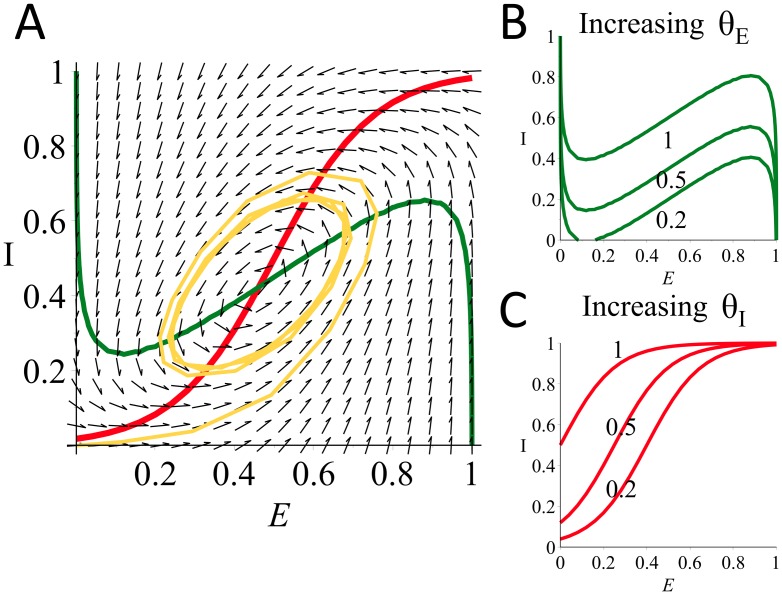
Analysis of the system's nullclines. A: phase plane of the system for *θ_E_* = 0.7, *θ_I_* = 0. E-nullcline (Equation (5)) (light grey), I-nullcline (Equation (6)) (black), trajectory beginning at E = 0,I = 0 (mid grey), vector field (black arrows). Intersection of nullclines corresponding to equilibrium at E = 0.46, I = 0.42. The eigenvalues of the system at this point are *λ_1_* = 1.16+2.64i, *λ_2_* = 1.16−2.64i, hence this equilibrium is unstable and trajectories converge to a limit cycle. B: effect on the E-nullcline of increasing input to the E population (*θ_E_*) from 0.2 to 1. C: effect on the I-nullcline of increasing input to the I population (*θ_I_*) from 0.2 to 1.

The I-nullcline (Equation (6)) has a typical sigmoidal shape; the E-nullcline (Equation (5)) takes the shape of an inverse sigmoid function. These two nullclines are plotted in black and light grey respectively in Figure 2A. The parameter values used to generate this figure are such that the nullclines only cross at a single point, forming the only equilibrium of the system. Due to the characteristic shape of the two nullclines it is possible for one, three or five intersections (i.e. equilibria of the system) to exist.

Adjusting the weight parameters and the *β* parameter in the model alters the steepness of the slopes of the nullclines. In particular, *w_EE_*, *w_EI_* and *β* can effect whether the E-nullcline has the characteristic ‘S-shape’ shown in Figure 2A (with two turning points) or whether it is an always decreasing function resembling a sigmoid rotated 90° counter-clockwise about its inflection point. In the latter case it is only possible for the two nullclines to cross at a single point. When the system's nullclines intersect in this fashion, i.e., at a point where the E-nullcline is decreasing and the I-nullcline is increasing, then the equilibrium that is formed is always stable. This can be shown by considering the system's Jacobian, which is evaluated at the equilibrium point (E*, I*):
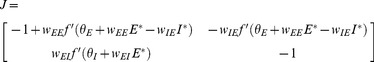
(7)


Notice that the sign of all the terms in the Jacobian is fixed, with the exception of the first term. It is known that an equilibrium point will be stable if the trace (*tr*) of the Jacobian (equivalent to the top left term plus the bottom right term in (7)) is negative and the determinant (Δ) (equivalent to the product of the top left and bottom right terms minus the product of the bottom left and top right terms in (7)) is positive [56]. This follows from the ability to formulate analytical expressions for the eigenvalues of the system as a function of *tr* and Δ (see [56] for details of this derivation):
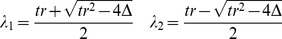
(8)


When *tr* is negative and Δ is positive both eigenvalues will have negative real parts and hence that point in the phase plane will be a stable fixed point. In our system this requirement will always be satisfied when the first term of the Jacobian is negative; since the bottom right term is always negative, when the first term is negative the sum of these two terms (i.e. *tr*) will always be less than zero, whilst Δ will always be positive. The first term of the Jacobian will always be negative if *w_EE_*<1, because the slope of the sigmoid function never exceeds 1 for our choice of *β* = 4. Thus if *w_EE_*<1 the fixed point will be stable. When *w_EE_*≤1 we can also be sure that this is the only fixed point in the system; following Wilson and Cowan [53], consider when the gradient of the E-nullcline at its inflection point (E = ½) is less than zero, this produces Equation (9):
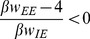
(9)For the choice of *β* = 4, if *w_EE_*≤1 Equation (9) is satisfied and the E-nullcline is an always decreasing function, whilst the I-nullcline is always increasing, therefore they can only intersect at a single point.

The gradient of the E-nullcline is equivalent to the quotient formed by dividing the first term in the Jacobian (Equation (7)) by the top right term and multiplying the result by −1 (see Mathematical Appendix for details of this derivation). Since the top right term in the Jacobian is always negative the gradient of the E nullcline will be negative when the first term in the Jacobian is negative and positive when the first term is positive. So the gradient of the E-nullcline is closely tied to the stability of any fixed points; when the gradient is negative so is the first term in the Jacobian and so the fixed point will be stable (*tr* is negative, Δ is positive and both eigenvalues have negative real parts as we have previously shown). When the gradient of the E-nullcline is positive so is the first term in the Jacobian and the fixed point may not be stable (dependent upon the parameter values which make up the Jacobian).

Any limit cycles that exist in a planar system must enclose at least one fixed point [56] and in the case of only one stable fixed point one cannot guarantee the existence of a limit cycle in the system's phase plane and hence that the system will generate intrinsic oscillations. Therefore, if the parameters of the system are such that the two nullclines only intersect at one point then the non-negative gradient of the E-nullcline at that point is a necessary condition for the system's only equilibrium to be unstable and for the system to produce oscillations as it follows a limit cycle trajectory around that unstable point (an example of this situation is given in Figure 2A).

## Results

### Dependence of oscillations on the constant input to E population

**Figure 3:**
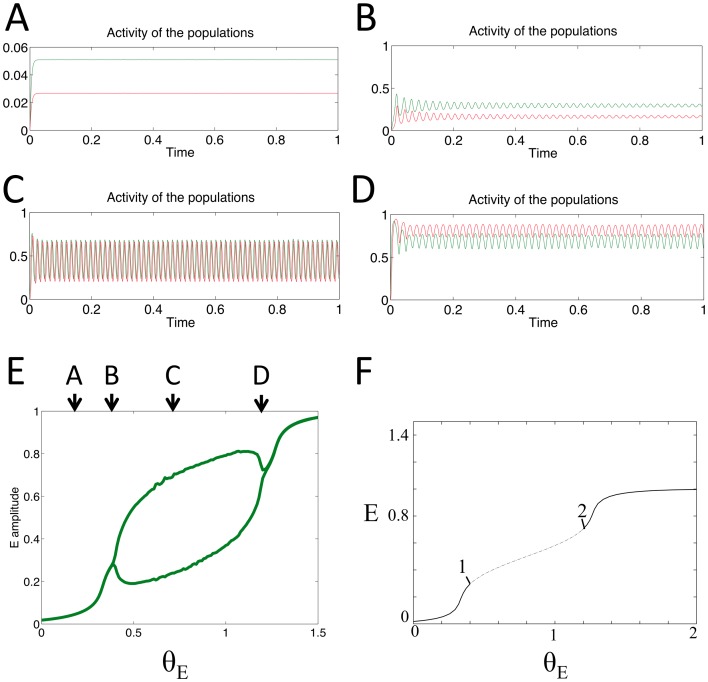
Behaviour of the model for a range of constant inputs, received by the E population only. Pictures A–D: output activity of the two populations E (grey) and I (black) when receiving constant input to E population of (A) 0.2, (B) 0.4, (C) 0.7 and (D) 1.18. E: maximum and minimum values of the E population's output activity are plotted (grey line) in order to display the region where these values differ and oscillations appear. Arrows indicate examples A–D. F: bifurcation diagram generated by continuation of the model's steady state equilibrium (*θ_E_* = *θ_I_* = 0, initial conditions: E = I = 0, equilibrium reached: E = 0.0181, I = 0.0207). Labelled points: (1) Hopf bifurcation, *θ_E_* = 0.399974, (2) Hopf bifurcation, *θ_E_* = 1.199932.

Here we report simulations in which we subjected the model to increasing levels of constant input to the E population, in order to verify that for certain values the system behaves as an oscillator. Both the E and I populations started with an initial value equal to zero. Some examples of the results of these simulations are shown in Figure 3. For low values of the input to the E population, *θ_E_*, the activity level of both populations converges to a steady state value (Figure 3A). However at a critical value of *θ_E_* both E and I begin to show oscillatory behavior (Figure 3B). As *θ_E_* is increased, the frequency of oscillations increases (Figure 3C). Further increase in *θ_E_* results in a decrease in the oscillations' amplitude and frequency (Figure 3D) and increasing *θ_E_* past a second critical value results in the two populations converging to a steady-state equilibrium once again. This appearance and disappearance of oscillatory behavior as *θ_E_* is varied is summarized in Figure 3E. Both the amplitude and the frequency of the oscillations are dependent on the value of *θ_E_*.

In order to confirm the bifurcation mechanism underlying this change in the dynamical behavior of the system and to determine the values at the bifurcation points, we conducted a continuation analysis of the model (Equation (1)) using the continuation software AUTO [57]. Continuing the initial steady state equilibrium which the populations reach for *θ_E_* = *θ_I_* = 0, using *θ_E_* as the continuation parameter, produced the bifurcation diagram shown in Figure 3F. This diagram demonstrates that a Hopf bifurcation point precedes the appearance of oscillations in the system (a Hopf bifurcation refers to the point at which the complex conjugate eigenvalues evaluated at an equilibrium of the system simultaneously change the sign of their real parts, leading to a corresponding change in the stability of that equilibrium). The disappearance of oscillations occurs in a similar way, following a Hopf bifurcation. Oscillations occur in the region between these two points, where only a single unstable equilibrium exists (as suggested by the Poincaré-Bendixson theorem and the presence of a trapping region in the system's phase plane). Although for the parameters we used the oscillations appeared through a Hopf bifurcation, for larger values of *w_EE_* they can appear through different bifurcations as discussed by Onslow [58].

### Generation of PAC via oscillatory input to E population

**Figure 4:**
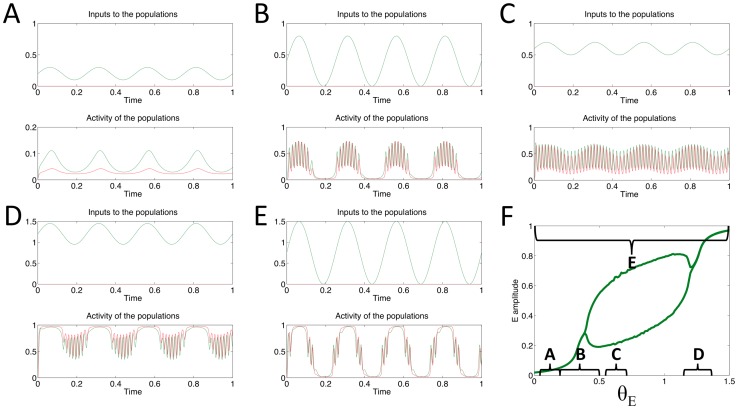
Behaviour of the model for a range of oscillatory inputs, received by the E population only. A–E: top panel shows the theta frequency oscillatory input to the E population; bottom panel shows the output of the E (grey) and I (black) populations. F: maximum and minimum values of the E population's output activity are plotted (grey line) in order to display the region where these values differ and oscillations appear. Brackets indicate the extent of the theta frequency input's amplitude in each of the examples A–E.

In the simulations described in this section the model was subjected to a theta frequency input oscillation to the E population of varying amplitude and mean. The inputs used in a simulation are plotted in the top panels of Figures 4A–E, while the labels in Figure 4F summarize the ranges of the inputs used in panels A–E. Since there exists a bounded region of values of *θ_E_* that permit the system to act as an oscillator and produce oscillations at its intrinsic frequency of 55 Hz (Figure 4F), this theta frequency input can move the system in and out of this region periodically. The result is gamma frequency oscillations phase-locked to different phases of the theta frequency input oscillation depending on the amplitude of the input. Depending on where the maximum and minimum values of the theta frequency input to the E population fall in relation to the bounded region for oscillations (Figure 4F) coupling to different phases of the input oscillation can be observed.

If the oscillatory input to E is of low amplitude (below the critical value at which the model produces intrinsic oscillations (*θ_E_CR1_* = 0.399974, Figure 3F, point 1) then the E population will tend to produce small amplitude oscillations of the same frequency (Figure 4A) as the input. The I population produces comparatively smaller amplitude oscillations at this frequency. However, if the input to E is such that its peak is above the critical value for the appearance of oscillations then the model exhibits PAC. Gamma frequency oscillations are able to occur around the peak phase of the theta input and appear to be nested within the slower input rhythm (Figure 4B). If the peak of *θ_E_* is above the critical value for the disappearance of oscillations (*θ_E_CR2_* = 1.199932, Figure 3F, point 2), but its minimum is above *θ_E_CR1_*, then the opposite qualitative type of PAC occurs; the high frequency oscillation occurs around the trough of the oscillating input (Figure 4D).

If the theta frequency *θ_E_* has its maximum and minimum values between *θ_E_CR1_* and *θ_E_CR2_*, then the two populations produce high frequency oscillations with close to constant amplitude but which contain a periodic variation in their mean value of the same frequency as *θ_E_* (Figure 4C). If the minimum value of *θ_E_* is below *θ_E_CR1_*, whilst the maximum is above *θ_E_CR2_*, then the E and I populations are able to produce gamma frequency oscillations locked to the ascending and descending phase of *θ_E_* (Figure 4E).

If the oscillatory input to the system is too fast relative to the system's intrinsic frequency then it could be difficult or impossible to observe one or more cycles of the faster intrinsic oscillation, since the system will move out of the oscillatory regime before a full intrinsic cycle is completed. It is the absolute value, not the frequency, of the input which determines the configuration of the nullclines and therefore whether the system is in an oscillatory regime and at what intrinsic frequency.

### Dependence of oscillations on the constant input to I population

**Figure 5:**
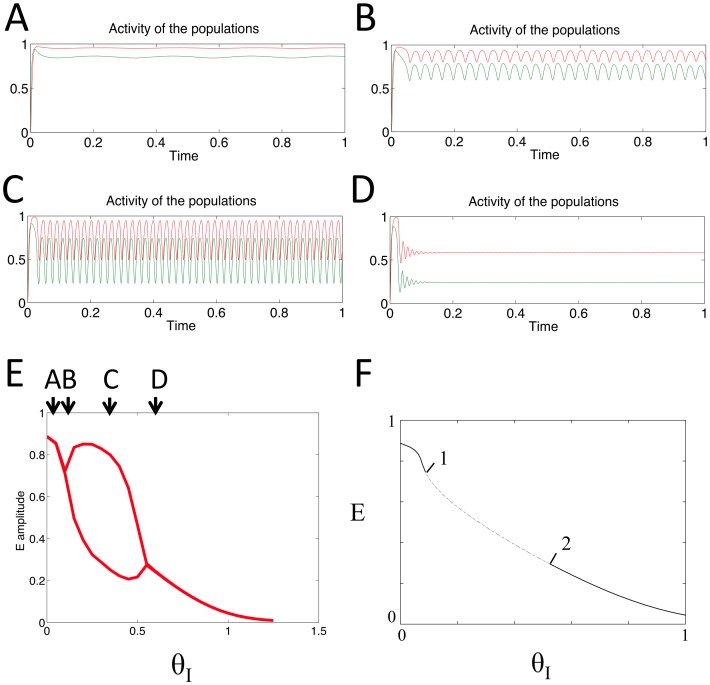
Behaviour of the model for a range of constant inputs, received by the I population (θ_E_ = 1.3). Pictures A–D: output activity of the two populations E (grey) and I (black) when receiving constant input to I population of (A) 0.05, (B) 0.12, (C) 0.4 and (D) 0.6. E: maximum and minimum values of the E population's output activity are plotted in order to display the region where oscillations appear. Arrows indicate examples A–D. F: bifurcation diagram generated by continuation of the model's steady state equilibrium (*θ_E_* = 1.3, *θ_I_* = 0, initial conditions: E = I = 0, equilibrium reached: E = 0.8873, I = 0.9568). Labelled points: (1) Hopf bifurcation, *θ_I_* = 0.105812, (2) Hopf bifurcation, *θ_I_* = 0.523650.

Initial simulations indicated that for the default parameter values we use it was not possible for the model to produce oscillations when input is given to the I population only. However for certain values of a constant input to E, varying a constant input to the I population will cause the system to move between a regime in which it produces intrinsic oscillations and one in which it does not. Figure 5 demonstrates this for a constant value of *θ_E_* = 1.3. The initial values of E and I are always zero. Figure 5E summarizes how increasing the value of *θ_I_* leads to first the appearance and then a gradual disappearance of oscillations in the system.

The oscillations that the system demonstrates as *θ_I_* is varied have a different shape to those seen when *θ_E_* was varied. Whereas in the latter case the activity level of the populations started at a low level and oscillations appeared as first an increasing then a decreasing activity level, in the case of varying *θ_I_* the activity levels of the populations start at a high level and oscillations appear as a decreasing, followed by an increasing activity level (see Figure 5B & C).

Continuation of the initial steady state equilibrium when *θ_E_* = 1.3 and *θ_I_* = 0 shows that very rapidly a stable equilibrium of the system becomes unstable through a Hopf bifurcation (at *θ_I_* = 0.105812). This marks the appearance of oscillations in the model. At *θ_I_* = 0.523650, the unstable equilibrium becomes stable, again through a Hopf bifurcation. After this point the eigenvalues defining the equilibrium are still complex but with negative real parts, therefore trajectories spiral towards the stable equilibrium, resulting in the damped oscillations seen in Figure 5D.

### Generation of PAC via oscillatory input to I population

**Figure 6:**
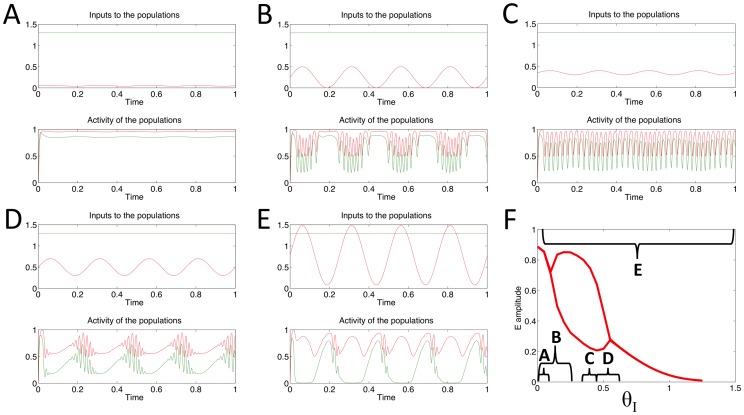
Behaviour of the model for a range of oscillatory inputs, received by the I population only (θ_E_ = 1.3). A–E: top panel shows the theta frequency oscillatory input to the E (grey) and I (black) populations; bottom panel shows the output of the E (grey) and I (black) populations. F: maximum and minimum values of the E population's output activity are plotted in order to display the region where oscillations appear. Brackets indicate the extent of the theta frequency input's amplitude in each of the examples A–E.

We forced the I population with a theta frequency oscillatory input, whilst the input to the E population was kept at a constant level of 1.3. Examples of the model's response to various amplitude oscillatory inputs are shown in Figure 6. Both the E and I populations started with an initial value equal to zero.

When the minimum and maximum values of the oscillatory input are below the critical value marking the appearance of oscillations (*θ_I_CR1_* = 0.105812, Figure 5F point 1), the E and I population produce low amplitude oscillations of the same frequency as *θ_I_* (Figure 6A). If the minimum value of *θ_I_* is below *θ_I_CR1_*, whilst the maximum value is above *θ_I_CR1_*, then during the peaks of *θ_I_* the system is able to produce intrinsic, high frequency oscillations, which appear nested within the slower input rhythm, demonstrating PAC (see Figure 6B). This situation is reversed if the minimum value of *θ_I_* is above *θ_I_CR1_* whilst the maximum value is above the critical value at which the Hopf bifurcation occurs (*θ_I_CR2_* = 0.523650, Figure 5F point 2). In this case the system would be expected to produce gamma oscillations during the trough phase of *θ_I_* (see Figure 6D). However, due to the gradient and shape of the ‘window’ for oscillatory behavior in this case (compare Figure 6F to Figure 4F), these oscillations appear to be more strongly coupled to the ascending phase of theta measured at the source of the input signal (Figure 6D top panel) and to the descending phase of theta measured in the local population (Figure 6D bottom panel).

If the minima and maxima of *θ_I_* occur between the two critical values *θ_I_CR1_* & *θ_I_CR2_*, then the system constantly produces high frequency oscillations, but with a mean value which varies with the same frequency as *θ_I_* (Figure 6C). If the minima and maxima are such that *θ_I_* moves periodically in and out of the region in which the system produces intrinsic oscillations, then the system attempts to produce high frequency oscillations on both the ascending and descending phases of *θ_I_* (Figure 6E), resulting in what appears to be gamma activity locked to the descending phase of every other cycle of a 8 Hz theta oscillation.

### Range of behaviour possible through variation of parameters

**Figure 7:**
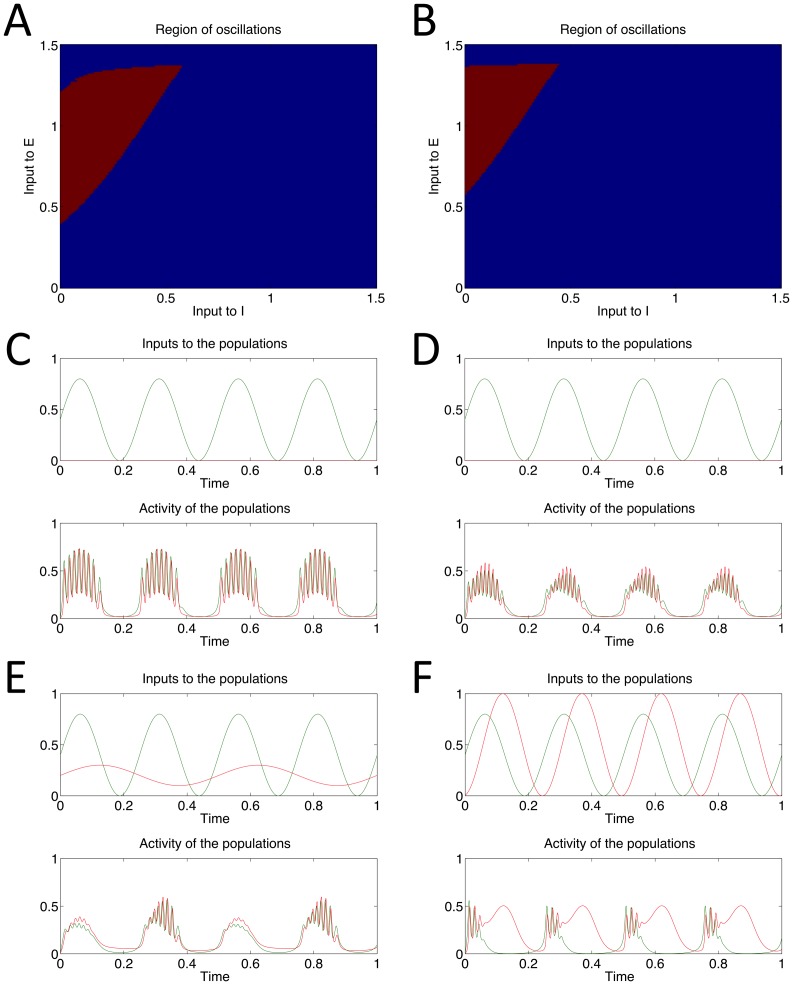
Range of behaviour of the model when weight and input parameters are varied. A: constant input values θ_E_ & θ_I_ were increased in incremental steps of size 0.01; if the resulting model activity was oscillatory the values are marked in red; if instead the E and I populations converged to constant values then the θ_E_ & θ_I_ values are marked in blue. B: same experiment as in A but for all simulations *w_EI_* = 2.5 (all other parameters take default values). The red region demonstrating oscillatory activity is smaller in comparison to that shown in A. C: example simulation showing theta-gamma PAC when all model parameters are set to default values. Modulation Index (MI) calculated on E's activity = 3.0. D: example simulations showing theta-gamma PAC when *w_EI_* = 2.5 (all other parameters take default values); in comparison to D the gamma activity is lower amplitude. MI calculated on E's activity = 2.333. E: simulation in which both *θ_E_* & *θ_I_* are oscillatory; *θ_E_* = 4 Hz, *θ_I_* = 2 Hz (amplitude and mean of the two input oscillations also differs, refer to top half of plot). Gamma activity appears locked to alternate theta cycles. F: simulation in which both *θ_E_* & *θ_I_* are oscillatory; both input oscillations have a frequency of 4 Hz but which both *θ_E_* lags *θ_I_* by 30° (amplitude and mean again differ slightly, refer to top half of plot). Whilst E demonstrates peak-locked PAC, I demonstrated gamma activity locked to the ascending phase of theta.

In order to summarize the constant values of *θ_E_* & *θ_I_* that produce oscillations in our system we ran multiple experiments in which we incrementally increased *θ_E_* & *θ_I_* with a step size of 0.01. The result is shown in Figure 7A, in which values of *θ_E_* & *θ_I_* that produce oscillatory behaviour are shown in red and values that converge to a steady-state equilibrium are shown in blue. This region of input values that leads to oscillatory behaviour in the model is also discussed in [58].

Figure 7B shows the results of the same multiple experiments that produced Figure 7A but with the parameter *w_EI_* in the model changed from 2 to 2.5. This results in a decrease in the size of the region for oscillatory behaviour (shown in red). In simulations this decrease in the size of the oscillatory region leads to smaller amplitude gamma oscillations occurring during each theta cycle, as can be seen when comparing Figure 7C (a simulation in which all parameters are set to their default values) and Figure 7D (a simulation in which *w_EI_* = 2.5, all other parameters take default values).

If both *θ_E_* & *θ_I_* are oscillatory and of different frequencies then a variety of different behaviours can be observed in the system. For example, if *θ_E_* = 4 Hz whilst *θ_I_* = 2 Hz, as shown in Figure 7E, then it is possible to observe gamma coupled to alternate cycles of the 4 Hz theta input rhythm. If the phase difference between an oscillatory *θ_E_* and an oscillatory *θ_I_* is non-zero then this can also produce various interesting behaviours, including gamma activity which is coupled to the ascending or descending phase of a theta frequency input, as shown in Figure 7F. In this simulation the two oscillatory inputs have the same frequency (4 Hz) but *θ_E_* lags behind *θ_I_* with a phase difference of 30°; this produces I population gamma activity that is locked to the ascending phase of a 4 Hz theta rhythm.

## Discussion

The results presented here demonstrate that PAC signals can arise when a slowly varying input to a neuronal population alters the dynamics of the local population-level network, such that at a certain phase of the input both populations produce intrinsic high frequency oscillations. For the network topology and parameters used here, the range of input values which lead to intrinsic oscillatory activity has both an upper and lower bound, enabling the slowly-varying input to push the system into this region either at its peak, during a trough or on both the ascending and descending phase (Figures 4,6 & 7F). When the input was received by the I population (whilst the E population received a simultaneous tonic input), it was possible to observe gamma activity which appeared to be locked to either the ascending or descending phase of the theta frequency input (Figure 6 D & E, ascending phase measured relative to theta frequency input itself and descending phase measured relative to the local activity theta oscillation). Coupling to the ascending phase was also demonstrated in the I population when both *θ_E_* & *θ_I_* consisted of 4 Hz theta oscillations but with a phase difference of 30° (Figure 7F).

Peak-locked [50], [51] and trough-locked [29], [36], [52] theta-gamma coupling have both been observed in vivo. There are also reports of gamma activity phase-locked to the ascending [36] and descending [39], [50], [51] phases separately, but as yet no reports of gamma phase-locked to both the ascending and descending phases of theta. There may be several reasons for this: one possibility is that biologically realized parameter values make it unfeasible for a slowly varying input to succeed in pushing the system through such a wide range of dynamics; a second possibility is that this form of PAC has been overlooked since only certain measures of PAC are able to detect bi-phasic coupling [59]. Finally, due to the changes in frequency experienced by the system as it passes through the Hopf bifurcations, this type of PAC might instead have been interpreted as the ‘concatenated rhythms’ phenomenon observed by Roopun et al. [60], who witnessed gamma (50–80 Hz) and beta2 (22–27 Hz) rhythms occurring alternately, as though locked to two different phases of a slower rhythm.

The functional relevance of coupling between these two rhythms remains to be confirmed but we would speculate that a general function for theta-gamma PAC is as a mechanism to integrate computations occurring on different time scales. For example, the period of a theta cycle corresponds well with the time taken to perform certain motor functions such as taking a step or sniffing [61]–[64] and might therefore be appropriate for processing the moment by moment environmental feedback received during such activities and structuring a sequence of events encountered within this typical time period. The much shorter period of gamma oscillations might similarly lend itself to grouping the more rapid (from the point of view of consciousness ‘spontaneous’) experience of several sensory inputs which all relate to the same percept [65], [66], for example processing visual scenes and recognizing objects within them whilst traversing a movement trajectory. Through a strict, phase-locked relationship between different frequencies, a hierarchy of computational processes that all converge to construct each momentary conscious experience might be effectively combined [18]–[21].

Our model is capable of reproducing other physiologically observed neural activity besides PAC. For example, for certain values of the weight and input parameters the model will produce gamma frequency activity on every other cycle of a theta frequency input (Figure 7E, also the I population in Figure 6E). This could potentially explain the observed theta-skipping behavior found in entorhinal neurons [67], [68], which appear to fire locked to LFP theta oscillations but only on every other cycle. The model is also capable of explaining the somewhat paradoxical result that low concentrations of ketamine, a drug which is understood to block excitation of inhibitory interneurons in hippocampal regions [69], result in decreased theta power but increased gamma power recorded in the LFP and EEG of mice and humans [70], [71]. Assuming theta frequency input is received by the I population in our model (whilst the E population receives a tonic input), if the I population were diminished in its ability to respond to this input this could correspond to a smaller amplitude and/or a lower mean value of the input theta oscillation in the model. This could be sufficient to move the system from a regime in which it produces gamma oscillations periodically, locked to the trough of the theta input, into a regime in which the dynamic range of the input is always within the boundaries for producing gamma oscillations (Figure 6F). The result would be a persistent gamma oscillation with a small amplitude theta variation of its mean (see Figure 6C).

Two recent papers have demonstrated through computational models and via optogenetic stimulation of layer II medial entorhinal cortex that theta frequency input to a network of cells can induce theta-gamma coupled activity [72], [73], in-line with the model presented here. Our model is also able to shed light on why gamma oscillations appear to be so closely phase-locked to a particular phase of a theta frequency optogenetic stimulation in the results of Pastoll et al. [72]; since the network as a whole crosses a bifurcation point at a given level of driving input. Without crossing this bifurcation point gamma frequency oscillations are not produced as the resonant network rhythm, since no limit cycle exists in the network's phase plane. The computational model results of Spaak et al. [73] suggest that differences in the model architecture between excitatory and inhibitory networks result in excitatory cells being more strongly biased than inhibitory cells by the incoming theta frequency activity, in accordance with physiological findings [74]. However, our model is able to suggest why their network as a whole requires some level of constant input to the excitatory population in order to generate PAC.

In contrast to the model presented here spiking neural networks are able to generate oscillations without recurrent E-E connectivity [75]. It is also the case that spiking networks of only inhibitory neurons can generate oscillations, a behavior which is not possible to observe in the firing rate model used here [76]–[78] but which has been observed in vitro [79]. PAC activity has also been demonstrated in purely inhibitory networks [43].

In order to compare our firing rate model with previous spiking models, the spiking model description must be reduced to an equivalent rate model description by assuming that synaptic dynamics are slow compared to the dynamics that transform synaptic input into firing rate [80]–[83]. This separation of time scales exists due to effective delays incorporated into spiking models by postsynaptic currents that persist for a period of milliseconds and would correspond to the incorporation of explicit time delays into the rate model discussed here; this could potentially enable our model to produce intrinsic oscillations without the current requirement for E-E connectivity. That a purely inhibitory population of neurons that exhibits a time delay on their recurrent connections can produce oscillatory behavior is explored in detail in [58], [76]. A detailed comparison between this firing rate model and previous spiking network models remains as a potential avenue for future work, however we note that several recent studies have demonstrated good agreement between spiking neural networks and their equivalent rate model descriptions [84], [85].

Future work will look to compare our firing rate framework more closely with biophysically realistic network models, in order to benefit from both an understanding of how nuances in connectivity and synapses can create behavior which breaks from the predictions of the mean field model and to use insights gained from mathematical analysis of the mean field model to choose which parameters to vary in order to witness bifurcations and new network attractor states.

### PAC and learning

There is evidence that the strength of theta-gamma PAC increases with learning over several days whilst rats are performing an item-context association task [37]. This could correspond either to an increasing signal-to-noise ratio, as more local populations engage in the same behavior demonstrated by our model, or to an increase in the window of input values that generate intrinsic oscillations in our model (window shown in Figures 3E & 5E; a comparative increase in this window occurs when parameter *w_EI_* is decreased from 2.5 to 2 – see Figure 7A&B). The latter could also be combined with an increase in the amplitude of the low frequency modulating input. If the window for intrinsic oscillations were increased in size then a low frequency modulating input with appropriate mean and amplitude could produce more high frequency cycles within each low frequency cycle, as well as high frequency cycle with a larger amplitude (Figure 7C & D), leading to a stronger high frequency signal being detected. Since this higher frequency signal always occurs at the same phase of the lower frequency oscillation, this would be detected as stronger theta-gamma PAC by the modulation index measure used in [37] (MI calculated on the E population output in Figure 7C = 3.0, MI calculated on the E population output in Figure 7D = 2.333).

We have demonstrated that the size of the window for intrinsic oscillations can be varied through changes in the model's synaptic weight parameters; this could occur in vivo via either long-term potentiation or depression during learning. The effect that changing each individual weight parameter has on the shape of the model's nullclines has been investigated in [58]. Short term, trial-length duration changes in PAC could be explained by dynamic variation in the controlling external input, in contrast to the longer term PAC variations brought about by changes in synaptic strength. Tuning of the various model parameters could affect not just the window of input values that produce PAC; it could also alter the intrinsic frequency response of the system. An increase in this frequency would make it possible to fit more gamma cycles within a theta cycle. This would correspond to an increased ability to store items according to the Lisman & Idiart scheme [23]. It is also the case that a decrease in the frequency of the theta input would allow more gamma cycles to fit within a theta cycle, increasing storage capacity (theta activity has been been shown to decrease in frequency in human subjects when they are asked to maintain more items in working memory, possibly indicating the need to allow more gamma cycles to nest within each theta cycle [86]).

### Relationship to empirical hippocampal data

Recurrent connections between excitatory and inhibitory cells in the hippocampus are well established and their interaction is understood to produce oscillatory activity. However, there is debate over the role of recurrent connections between excitatory cells in generating oscillatory activity, particularly at gamma frequencies [87].

Recurrent excitation is a common feature of neocortical microcircuitry [88] and the structure of our model is intentionally general in order to make it applicable to a variety of brain regions, not just the hippocampus. Recurrent excitation between pyramidal neurons is evident in regions such as prefrontal [89], visual [90] and barrel cortex in rats [91], regions that are also known to demonstrate gamma and theta-gamma-PAC oscillations [76], [92]. Specifically in the hippocampus, there is evidence for recurrent projections between excitatory cell types in regions CA1 [93] and CA3 [94] which have both demonstrated coupled theta-gamma PAC activity [27], [37]. Although the recurrent excitatory projections within CA1 are believed to be less numerous than those found within CA3, the effect of weak or sparse recurrent excitatory connections in local hippocampal regions may be amplified by the activity of astrocytes [95].

An alternative to the recurrent, positive feedback in E would be the introduction of synaptic transmission delays into the model (see [96] for example), delaying inhibition while E's activity increases at the start of the cycle and effectively amplifying the increase in E's activity. Whilst the topology and synaptic physiology of E-E and E-I connections in neural circuits vary across different brain regions, we chose to take advantage of the mathematical simplicity of a model based on recurrent E-E connections. However, insights from this model also apply to circuits with recurrent connections functionally substituted by synaptic transmission delays.

Considering the architecture of our model as representative of neural populations in the hippocampus, there is experimental support for theta frequency input from medial septum being received by both excitatory and inhibitory hippocampal neurons: alongside glutamatergic projections from septal regions to hippocampal pyramidal cells [97], there is also evidence for cholinergic projections which target hippocampal pyramidal cells and interneurons and GABAergic projections that exclusively target interneurons [98]–[101]. The GABAergic projections to hippocampal interneurons could provide the excitatory theta frequency input used in our model by way of their disinhibitory effect. A study by Wulff et al. [102], which ablated synaptic inhibition in hippocampal parvalbumin-positive (PV) interneurons, found that the loss of inhibition of these neurons led theta-gamma coupling to change, from gamma locked to the peak of a strong theta oscillation to gamma which occurred at all phases of a weaker theta oscillation (i.e. gamma activity with a theta-varying mean, as in Figure 4C). This could be explained in our model if the loss of inhibition had the same affect as increasing the excitatory drive in the model (*θ_E_*), moving the system's dynamic range in such a way that it fits entirely within the window in which intrinsic gamma oscillations are produced (Figure 4F).

### A general circuit for PAC occurring in other brain regions

The general neural circuit presented here might also function as a model for PAC activity occurring in a variety of other brain regions, which receive a low frequency input and produce higher frequency activity phase-locked to a particular phase of that input. Theta-gamma PAC for example has also been reported in prefrontal cortex [74] and entorhinal cortex [103]. Whilst this activity could result from the interaction of gamma activity with locally generated theta oscillations, it could also reflect cross-structural interactions, since both structures receive theta frequency input from the medial septum [104]–[106] and prefrontal cortex also receives theta frequency input from hippocampus [107]. Populations of neurons that form this characteristic circuit in prefrontal and entorhinal regions would produce gamma frequency activity phase-locked to the theta frequency input. This temporal relationship might facilitate a shared representation of information between the sending and receiving structures, aiding interpretation of this combined information downstream or facilitating computation occurring in a feedback loop between the two structures.

## Conclusions

We have presented a firing rate model of two neural populations, which is capable of producing high frequency oscillations that are locked to certain phase(s) of a lower frequency oscillatory input. This pattern of activity resembles the PAC activity that has been recorded in electrophysiological data. The amplitude, frequency and phase-locking characteristics of the PAC activity generated are dependent on the strength of the connections in the model and on the amplitude and mean value of the low frequency input signal. The input signal is responsible for shifting the system periodically across a bounded region of input values corresponding to a regime in which the system produces intrinsic high frequency oscillations. Within this bounded region, the limits of which coincide with Hopf bifurcation points, there exists a single, unstable equilibrium in the system's phase plane, surrounded by a limit cycle, to which all trajectories of the system converge. Therefore within this region the system behaves as an oscillator and generates oscillations of characteristic frequency. It is possible to tune the parameters of the model to produce different frequencies of activity phase-locked to different phases of the input rhythm, a feature which was not present in previous PAC models.

Our model suggests that if the phase of slow oscillations in one area appears to modulate the amplitude of fast oscillations in another area then it is possible that this first area sends a slow frequency oscillatory input to the second area, which constrains the timing of the fast oscillations produced there. This could in turn constrain the firing of individual neurons in that local population [77], [84] and therefore prove functionally significant for computational processes occurring collaboratively between the sending and receiving regions, as well as regions downstream.

This work differs from previous models capable of generating PAC in that its general, canonical circuit basis could apply to a variety of brain regions. It has the ability to explain how PAC activity might arise from population level mechanisms and how the system might be varied in order for the high frequency activity to lock to different phases of the low frequency input oscillation. Future work will ideally look at comparing this model both with experimental data and with detailed multi-single neuron models, to examine its applicability to various neural circuits, the ensuing parameterizations and how many neurons are required to make up a representative population. Useful comparisons might also be drawn in future between the general features of this population level model and models of hyperpolarizing and depolarizing currents in single neuron models, in order to explore PAC activity observed in individual cell membrane potential traces [72]. Since temporal relationships such as PAC occurring at the population level might bias single neuron firing and influence neuronal processing, modeling and in vivo studies of this phenomenon are likely to be fruitful directions for future research.

## Mathematical Appendix

The gradient of the ‘x’-nullcline in a planar nonlinear system is equivalent to the quotient formed by dividing the top left term in the system's Jacobian by the top right term and multiplying the result by −1. The gradient of the ‘y’-nullcline is equivalent to the bottom left term divided by the bottom right term and multiplied by −1. This can be shown by first acknowledging that in linearizing about a fixed point we are approximating a nonlinear system by an equivalent linear description at that point and secondly by considering the general form of two coupled linear differential equations (Equation (10)):
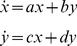
(10)The Jacobian of this system is:
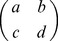
(11)The nullclines are:
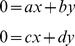
(12)The two nullcline equations in (12) can be rearranged to give two equations of straight lines:
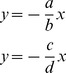
(13)This demonstrates the relationship between the gradients of the nullclines and the terms in the Jacobian when we linearize about a fixed point.
